# Genetic polymorphisms as predictors of the response of hepatocellular carcinoma patients to doxorubicin chemotherapy: a genome-wide association study

**DOI:** 10.3389/fphar.2025.1604473

**Published:** 2025-06-04

**Authors:** Sireen Abdul Rahim Shilbayeh, Naglaa F. Khedr, Mohammad A. Alshabeeb, Abdulmonem Ali Alsaleh, Abdalrhman Hamdan Alanizi, Omnia A. Abd El-Baset, Rehab H. Werida

**Affiliations:** ^1^ Department of Pharmacy Practice, College of Pharmacy, Princess Nourah bint Abdulrahman University, Riyadh, Saudi Arabia; ^2^ Biochemistry Department, Faculty of Pharmacy, Tanta University, Tanta, Egypt; ^3^ Pharmaceutical Analysis Center, King Abdullah International Medical Research Center (KAIMRC), King Saud bin Abdulaziz University for Health Sciences (KSAU-HS), Ministry of National Guard Health Affairs (MNGHA), Riyadh, Saudi Arabia; ^4^ Blood and Cancer Research Department, King Abdullah International Medical Research Center (KAIMRC), King Saud bin Abdulaziz University for Health Sciences (KSAU-HS), Ministry of National Guard Health Affairs (MNGHA), Riyadh, Saudi Arabia; ^5^ Department of Pharmaceutical Care Services, Medical Affairs, King Abdullah Bin Abdulaziz University Hospital, Riyadh, Saudi Arabia; ^6^ Clinical Pharmacy and Pharmacy Practice Department, Faculty of Pharmacy, Egyptian Russian University, Cairo, Egypt; ^7^ Department of Clinical Pharmacy and Pharmacy Practice, Faculty of Pharmacy, Damanhour University, Damanhour, Egypt

**Keywords:** genome-wide association study, hepatocellular carcinoma, doxorubicin, transarterial chemoembolization, array genotyping

## Abstract

**Background:**

Hepatocellular carcinoma (HCC), a leading cause of cancer-related mortality, is commonly treated with doxorubicin (DOX). However, its effectiveness varies significantly among patients.

**Aim:**

The present study aimed to identify potential genetic variants affecting the response of HCC patients to DOX.

**Methods:**

78 patients with HCC who received DOX via transarterial chemoembolization (TACE) technology were selected. DNA was extracted from blood for genome-wide genotyping using the Applied Biosystems™ Axiom™ Precision Medicine Diversity Research™ Array. Genetic data were analysed using Axiom™ Analysis Suite software v5.2.

**Results:**

Six hits in five genes [AK3 (rs378117), TRPM3 (rs1329774 and rs4745058), CDH4 (rs2427043), LINC00504 (rs76228864), and GRIN2D (rs76754767)] were associated with a risk of tumour progression, whereas variants in HPGD (rs45593131) and RC3H2 (rs2792999) were suggested as protective factors. rs8038528 in the PCSK6 gene was categorized as a low-response variant associated with an unsatisfactory reduction in α-fetoprotein (AFP) levels after DOX chemotherapy (P = 6.82 × 10^−5^). In contrast, three SNPs (rs1998853, rs12440990, and rs4774596) located within two genes (NPAS3 and DMXL2) were identified as predictors of good response rates to the treatment, as AFP levels were reduced by ≥ 20%. Death incidents showed associations with five SNPs that reached p ≤ 5.0 × 10^−8^; four of these are located within the DENND1B, LOC107986086, TMEM169, and RNF152 genes.

**Conclusion:**

These findings support the incorporation of pharmacogenomic testing into clinical practice for HCC therapy, paving the way for customized treatment methods that may improve therapeutic efficacy and patient outcomes. Future research is needed to replicate these genetic connections.

## 1 Introduction

According to the International Agency for Research on Cancer (IARC) and partners, hepatocellular carcinoma (HCC) is the most common subtype of primary liver cancer. HCC accounts for 80% of all primary liver cancers globally (Rumgay et al., 2022) and is the third leading cause of cancer-related mortality (Konyn et al., 2021). However, the incidence and risk factors for HCC vary across different regions and countries. The highest rates were reported in eastern Asia, northern Africa, and southeastern Asia (Rumgay et al., 2022). In the Arab world, HCC represents a major health problem, especially in Egypt, which has the highest number of cases (Abudeif, 2019). The main risk factor for HCC in Egypt is infection with hepatitis C virus (HCV), which affects approximately 15% of the population (Abudeif, 2019). In other Arab countries, such as Saudi Arabia, the United Arab Emirates (UAE), and Morocco, hepatitis B virus (HBV) is the predominant cause of HCC (El-Kassas and Elbadry, 2022). Other factors that contribute to HCC in the Arab region include obesity, diabetes, alcohol consumption, and nonalcoholic steatohepatitis (NASH) (Hashim et al., 2021).

The American Association for the Study of Liver Diseases (AASLD) developed evidence-based practice guidelines, including therapeutic and preventive aspects of care. These guidelines consider changing epidemiological patterns, with an increasing proportion of cases related to nonviral aetiologies (Singal et al., 2023). The Barcelona Clinic Liver Cancer Staging and Treatment Allocation (BCLC) categorization divides HCC into five stages: very early, early, intermediate, advanced, and terminal (Li et al., 2023). Early-stage HCC is best managed by curative treatment, which includes surgical resection, ablation, or transplantation. Patients with intermediate-stage disease, particularly unresectable HCC, often receive palliative therapy with locoregional treatment, whereas systemic treatment, including chemotherapy, tyrosine kinase inhibitors, or immunotherapy, is reserved for patients with advanced disease (Singal et al., 2023).

One method of locoregional treatment is transarterial chemoembolization (TACE), which involves injecting a chemotherapy drug (such as doxorubicin [DOX]) mixed with an embolic agent (such as ethiodized oil or microspheres) into the hepatic artery that supplies blood to the tumour. This localized administration causes an infarct and subsequent necrosis of the tumour. This treatment approach is preferred over systemic chemotherapy, as the drug can directly target cancer cells and block their blood supply while minimizing exposure to the rest of the body (Bessar et al., 2021). Additionally, in theory, TACE provides better groundwork for subsequent major surgery and likely prolongs long-term survival if it is initiated at an early stage of HCC. Over the last decade, TACE has been shown to be a promising technique for the treatment of HCC in terms of overall survival (OS) or the tumour response, with minimal adverse events (Chen et al., 2017; Han et al., 2019). However, it is still accompanied by several limitations and challenges, such as determining the optimal dose and selecting the most effective chemotherapy drug (epirubicin, DOX, cisplatin, or adriamycin).

DOX is the most common cytotoxic drug employed in TACE (Bessar et al., 2021; Tam, 2013) as a mono-drug or in combination with chemotherapy (Fan et al., 2017; Liu et al., 2015; Feng et al., 2018) at fixed or variable doses depending on the patient variables (body surface area and weight) and the size of the tumour (Bessar et al., 2021). Although it can be infused systemically, this route is avoided because of its severe side effects, especially cardiac toxicity (van der Zanden et al., 2021). To date, the exact mechanisms by which DOX mediates cancer cell dysfunction or death are not fully understood (Micallef and Baron, 2021). However, they are mostly attributed to the ability of DOX to intercalate into tumour cell DNA, inhibit topoisomerase II, disrupt mitochondrial function, and potentiate free radical generation and oxidative damage, in addition to its immunomodulatory role (Kciuk et al., 2023). Some of these mechanisms are also linked to multiorgan toxicity induced by DOX due to intercalation in the DNA of healthy cells, particularly when it is infused systemically (van der Zanden et al., 2021; Pugazhendhi et al., 2018). Furthermore, cumulative evidence from *in vivo* and *in vitro* studies examining the chemical structure and metabolism of DOX has suggested alternative mechanisms of anticancer activity (Bisht et al., 2024).

The Pharmacogenomics Knowledge Base (PharmGKB) database has built integrated complex pharmacokinetic (PK)–pharmacodynamic (PD) pathways illustrating the fate of this drug molecule in the human body (Thorn et al., 2013), which were deemed beneficial for pharmacogenomic (PGx) research attempting to define how genetic variations may explain interindividual and interethnic variability in DOX PKs and PDs and hence affect the clinical response to DOX (Thorn et al., 2013). In summary, DOX is transported into cells by the solute carrier family (SLC) of membrane proteins (e.g., SLC28A3 and SLC22A16). A significant fraction (approximately 50%) of the unchanged drug undergoes efflux from body cells via transporters of the ATP-binding cassette family (ABCB1, ABCC1, ABCG2, and ABCC2) and RA1-binding proteins (RABLP1). The remaining proportion of drugs in cells undergoes metabolism, mainly to doxorubicinol and DOX-semiquinone radicals; both metabolites have lower antineoplastic activity, but they are more prone to induce cardiotoxicity (Kassner et al., 2008). A carbonic anhydrase isoform (CBR1) was found to be its main metabolic carrier to liver cells (Kassner et al., 2008; Gonzalez-Covarrubias et al., 2009), whereas an oxidoreductase isoform (AKR1A) was shown to be its most important carrier into heart tissues (Mordente et al., 2003).

To date, studies of several candidate genes have examined the impact of germline genetic polymorphisms in selected key genes involved directly in DOX PK/PD pathways to determine their individual associations with effectiveness and safety in multiethnic groups of patients with several types of cancer, such as breast neoplasms (Nyangwara et al., 2024; Ruiz-Pinto et al., 2018; Hertz et al., 2016; Lal et al., 2016; Faraji et al., 2016), non-Hodgkin lymphoma (Wojnowski et al., 2005), osteosarcoma (Windsor et al., 2011), soft tissue sarcoma (Gelderblom et al., 2014), or acute lymphoblastic leukaemia (Gándara-Mireles et al., 2021).

However, the associations obtained from these studies are often inconclusive, limited to specific cancer complications (such as cardiotoxicity), and confined to certain ethnogeographic groups (Nyangwara et al., 2024; Ruiz-Pinto et al., 2018; Hertz et al., 2016; Lal et al., 2016; Faraji et al., 2016). Therefore, the identified PGx markers were rated based on the PharmGKB clinical annotation scoring system as level 3, which indicates a lower level of evidence based on a single study or several studies that failed to replicate the association or were based on preliminary evidence (Whirl-Carrillo et al., 2012; Whirl-Carrillo et al., 2021). Therefore, in terms of clinical significance, further replication in larger cohort studies involving diverse ethnic populations is needed. Consequently, these constrained findings have hindered the development and implementation of unified guidelines for routine pharmacogenomic testing in oncology clinical practice employing DOX chemotherapy (Nagy et al., 2020). In routine practice, the pharmacogenetic profile of DOX was rarely examined under the condition its usage via TACE technology in HCC patients (Liang et al., 2016). This finding motivated us to conduct the present study. The current pharmacogenomic study aimed to determine potential variants that predict the effectiveness and outcomes of DOX chemotherapy mediated by TACE in HCC patients.

## 2 Materials and methods

### 2.1 Study design and patients

This prospective study was conducted between August 2022 and December 2023 in qualified oncology departments that specialize in the management of hepatocellular carcinoma (HCC) at university hospitals. The Institutional Review Board (IRB) of Princess Nourah University (PNU) in Riyadh, Saudi Arabia, approved all study procedures (IRB Log Number: 23–0177) in compliance with recognized ethical standards.

Initially, 224 consecutive patients with HCC, both male and female, were screened for potential inclusion in the study. Each patient underwent a comprehensive review of their medical history and laboratory results. The eligibility criteria required participants to be aged 18 years or older and to have a confirmed diagnosis of intermediate- or advanced-stage HCC, as verified by histological, radiological, or pathological analysis following the guidelines of AASLD (Singal et al., 2023). Some patients were excluded after staging due to various reasons as described in the Flow chart (Supplementary Figure S1).

Enrolled patients received DOX through TACE technology based on expert opinion, as their tumors were deemed unresectable or they were not candidates for radiofrequency ablation. Patients were excluded from the study if they met any of the following criteria: age over 75 years, presence of portal vein thrombosis, white blood cell count less than 3 × 10^9^/L, platelet count below 50 × 10^9^/L, serum bilirubin level greater than 3 mg/dL, serum creatinine concentration exceeding 1.5 mg/dL, history of co-occurring illnesses, or a diagnosis of other types of cancer.

All patients provided written informed consent before enrollment. Primary data were collected from medical records, including demographic and clinical information, baseline laboratory values, and concurrent medication use. The data were entered anonymously into an electronic annotated data entry program (Harris et al., 2019).

In accordance with a standard TACE treatment protocol, each patient was intra-arterially infused with a combination emulsion containing two medications: ethionized oil and DOX. For tumours with a diameter <5 cm, the ethionized oil dose was <5 mL. Tumours ≥5 cm in size were administered a maximum dose of 10 mL. The DOX dose ranged between 30 and 60 mg/m^2^, depending on the tumour size, extent, and blood supply.

### 2.2 Study oversight

This observational study conducted follow-up over 13 months to comprehensively capture clinical outcomes, specifically treatment efficacy. Triple pelvic abdominal computed tomography (CT) scans were conducted prior to and 1 month subsequent to TACE to detect recurrence and assess the necessity for an additional therapeutic cycle. Follow-up appointments were arranged for all patients, including those who attained a complete response (CR), to determine any adverse effects and to evaluate hematologic, renal, and hepatic function.

### 2.3 Clinical outcome definitions

To assess the efficacy of DOX delivered through TACE, we assertively utilized critical outcome measures: overall survival (OS), progression-free survival (PFS), and α-fetoprotein reduction. These metrics were meticulously defined comprehensively in previous studies (Lencioni and Llovet, 2010; Liu et al., 2019; Masior et al., 2023; Bruix, 2021), reinforcing the rigor of our, evaluation and the significance of our findings.

### 2.4 Genotyping method and quality control

On the first day of the patients’ visit, blood samples were collected into two 5 mL EDTA-containing tubes for genotyping. Genomic DNA was extracted from whole blood using the QIAsymphony SP automated extraction system and a QIAsymphony^®^ DSP DNA Midi Kit, following the manufacturer’s instructions (Qiagen). A Nanodrop spectrophotometer was utilized to determine the concentration and purity of the extracted DNA.

The extracted genomic DNA samples were then amplified using multiplex PCR with a QIAGEN Multiplex PCR Kit, as per the manufacturer’s instructions (Qiagen). This amplification step allowed for high-resolution genotyping of highly homologous regions of the genome.

Following amplification, genome-wide genotyping of the participants was carried out using the Applied Biosystems™ Axiom™ Precision Medicine Diversity (PMD) Research™ Array technology on the automated Applied Biosystems™ GeneTitan™ Multi-Channel (MC) instrument (Affymetrix Inc., Santa Clara, CA, United States). This Axiom Array solution provides comprehensive coverage of over 850,000 single-nucleotide polymorphisms (SNPs), insertions or deletions (indels), and copy number variants (CNVs), along with dense whole-genome coverage across diverse populations [catalogue identifier: 951961; Thermo Fisher Scientific, https://www.thermofisher.com/order/catalogue/product/951961/] (Thermo Fisher Scientific, 2024). It also includes a thorough analysis of over 5,000 pharmacogenomic (PGx) markers in more than 1,100 core and extended pharmacokinetic/pharmacodynamic (PK/PD) genes, evaluated at clinical annotation levels of evidence 1–4 (as established by PharmGKB). These genes influence the absorption, distribution, metabolism, and excretion (ADME) of commonly prescribed medications.

The generated genotyping profiles were analyzed using the Applied Biosystems™ Axiom™ Analysis Suite software version 5.2 (Thermo Fisher Scientific, Santa Clara, CA, United States). Markers corresponding to candidate genes with a genotyping call rate of less than 95%, minor allele frequency (MAF) lower than 0.01, or Hardy-Weinberg equilibrium (HWE) P-value less than 0.001 were excluded from the association analysis. Additionally, samples with a genotyping call rate below 93% were also excluded.

### 2.5 Selection of markers of interest

The Ensembl Variant Effect Predictor (VEP) tool was used to annotate and prioritize the identified markers based on their predicted effects (McLaren et al., 2016). The selected variants were located on known genes and characterized by their functional consequences for gene activity.

### 2.6 Statistical and bioinformatics analyses

PLINK version 1.90p 64-bit (16 April 2021) was used for the statistical analysis (www.cog-genomics.org/plink/1.9/), General Public Licence v3 (Purcell et al., 2007). A threshold P value <5 × 10^−8^ was used for genome-wide association study (GWAS) analyses as a strict significance point to identify potential loci (Witte et al., 1996). However, some signals with lower association signals (P < 5 × 10^−5^) were also suggested for variants with known functional consequences. The Kaplan–Meier analysis was performed to examine the long-term significance of the effects of the identified top hit genotypes on PFS and OS. Manhattan plots were generated using the R statistical package (qqman) version R-4.2.2. The association values shown in Manhattan plots are reported as–log10 P values. The haplotype analysis software tool version 1.05, prepared by Eliades and Eliades (2009), was used to determine the common significant haplotypes.

## 3 Results

### 3.1 Selection of axiom array markers

Approximately 70,000 variants did not exist in the genotyped cohort. Thus, the remaining 780,166 variants were tested and analysed in 81 HCC patients (59 males, 22 females); however, three samples were removed (Supplementary Figure S1) because of the low sample genotyping call rate (<93%). The average genotyping call rate in the remaining samples was 99.6%. Several genotyping quality control (QC) measures were applied; thus, 45,901 variants were removed because of a low variant genotyping call rate (<95%), 138 variants were removed because of the Hardy‒Weinberg exact test, and 258,664 variants were removed because of the minor allele threshold (<1%). The remaining 475,463 variants and 78 patients passed filters and QC measures (35 patients experienced progressive symptoms, 43 had no progression, 8 patients died, 34 had a good AFP response, and 41 had no response). The genomic inflation factor for all GWAS analyses was <1.05.

### 3.2 Patient characteristics

Table 1 shows the demographic and clinical data of HCC patients who experienced progression (symptomatic or radiographic) versus patients with no evidence of progression. The majority of the studied patients were males (71.8%), yet the male gender difference between the two tested groups (74.4% with no progression vs 68.6% of patients who experienced progression) was not statistically significant (P = 0.568). The mean age of the studied patients was 62.01 years (61.93 ± 6.47 vs 62.11 ± 6.69 years; P = 0.903). Additionally, baseline laboratory data (including albumin, haemoglobin, bilirubin, alanine transaminase (ALT) and aspartate transaminase (AST) levels) were not different between patients who experienced progression and their counterparts who did not have any evidence of progression. On the other hand, baseline AFP measurements were substantially lower in the nonprogression group than in the tumour progression group (mean = 15.5 ng/mL vs. 123.0 ng/mL, respectively; P = 0.009). The majority of the patients in both groups received TACE once (90.7% vs 94.3%, respectively; P = 0.55). Macroscopic vascular invasion and metastatic extension were more common among patients who experienced progression than among those who did not (P = 0.003 and 0.005, respectively). The other clinical characteristics of the patients were equally distributed, as shown in Table 1.

**TABLE 1:** The HCC cases with symptomatic or radiographic progression were compared to those without progression.

Demographic data	Total, n (%)	No progression, n (%) (n = 43)	Progression, n (%) (n = 35)	P Value
Sex				
Male	56 (71.8)	32 (74.4)	24 (68.6)	0.568
Female	22 (28.2)	11 (25.6)	11 (31.4)	0.568
Age (years)				
Mean ± SD	62.01 ± 6.53	61.93 ± 6.47	62.11 ± 6.69	0.903
Median (range)	61.0 (48–74)			
Lab. Investigations				
Albumin, g/dL, mean ± SD	3.64 ± 0.54	3.69 ± 0.53	3.57 ± 0.56	0.355
Haemoglobin, g/dL, mean ± SD	12.13 ± 1.90	12.00 ± 2.13	12.29 ± 1.60	0.491
Bilirubin, mg/dL, mean ± SD	1.01 ± 0.40	0.98 ± 0.34	1.06 ± 0.46	0.359
ALT, U/L, median (range)	38 (15–219)	39.50 (16–219)	35.00 (15–111)	0.608
AST, U/L, median (range)	50.50 (18–248)	51.50 (18–248)	50.00 (20–186)	0.988
AFP, ng/mL, median (range)	50.0 (0.63–36979)	15.55 (0.63–7710)	123.00 (3.7–36979)	0.009
TACE frequency				
1	72 (92.3)	39 (90.7)	33 (94.3)	0.554
2	4 (5.1)	3 (7)	1 (2.9)	0.412
3	2 (2.6)	1 (2.3)	1 (2.9)	0.883
Systemic anticancer therapy				
Sorafenib	8 (10.3)	3 (7)	5 (14.3)	0.290
Hormonal therapy	5 (6.4)	1 (2.3)	4 (11.9)	0.103
Systemic antiviral therapy	19 (24.4)	13 (30.2)	6 (17.1)	0.180
History of comorbidities and prognostic criteria				
Hepatitis C	73 (93.6)	41 (95.3)	32 (91.4)	0.482
Hepatitis B	3 (3.8)	1 (2.3)	2 (5.7)	0.439
Liver cirrhosis	49 (62.8)	28 (65.1)	21 (60)	0.642
Ascites	17 (21.8)	6 (14)	11 (31.4)	0.063
Macroscopic vascular invasion	17 (21.8)	4 (9.3)	13 (37.1)	0.003^a^
Extrahepatic spread	6 (7.7)	2 (4.7)	4 (11.4)	0.264
Child‒Pugh class				
A	55 (70.5)	32 (74.4)	23 (65.7)	0.402
B	21 (26.9)	11 (25.6)	10 (28.6)	0.767
C	2 (2.6)	0 (0)	2 (5.7)	0.112
Tumour characteristics				
BCLC stage				
0	8 (10.3)	7 (16.3)	1 (2.9)	0.052
A	33 (42.3)	17 (39.5)	16 (45.7)	0.583
B	37 (47.4)	19 (44.2)	18 (51.4)	0.524
HCC HFL				
1 HCC <2 cm carcinoma *in situ*	10 (12.8)	8 (18.6)	2 (5.7)	0.090
1 HCC or 3 nodules <3 cm	30 (38.5)	15 (34.9)	15 (42.9)	0.472
Multi nodular	38 (48.7)	20 (46.5)	18 (51.4)	0.666
Disease extension				
Liver only	64 (82.1)	40 (93)	24 (68.6)	0.005^a^
Metastatic	14 (17.9)	3 (7)	11 (31.4)	0.005^a^

Notes:

^a^ Significant p value <0.05.

The values are presented as the means ± SDs, medians (ranges), or numbers (percentages), as appropriate.

The data were analysed via the chi-square test, Mann‒Whitney U test or t-test, as appropriate.

Abbreviations: AFP, alpha-fetoprotein; ALT, alanine transaminase; AST, aspartate aminotransferase; BCLC, barcelona clinic liver cancer staging and treatment allocation system; bilirubin, total bilirubin; HFL, hepatic focal lesion; TACE, transcatheter arterial chemoembolization.

A multivariate Cox proportional hazards model with a forward likelihood ratio revealed that the presence of metastatic disease extension was the most significant predictor of shorter OS, with an HR of 8.36 (95% CI 1.1–63.5, P = 0.04), while hepatitis B etiology was the strongest predictor of shorter PFS (HR: 5.04, 95% CI 1.26–20.1, P = 0.022). In addition, combined genetic and nongenetic multivariate association analyses revealed that metastatic disease extension (P = 0.015) was the only non-genetic predictor factor modulating the impact of DOX on OS in conjunction with other genotypic variants.

### 3.3 Genetic associations with progression and PFS

**FIGURE 1:**
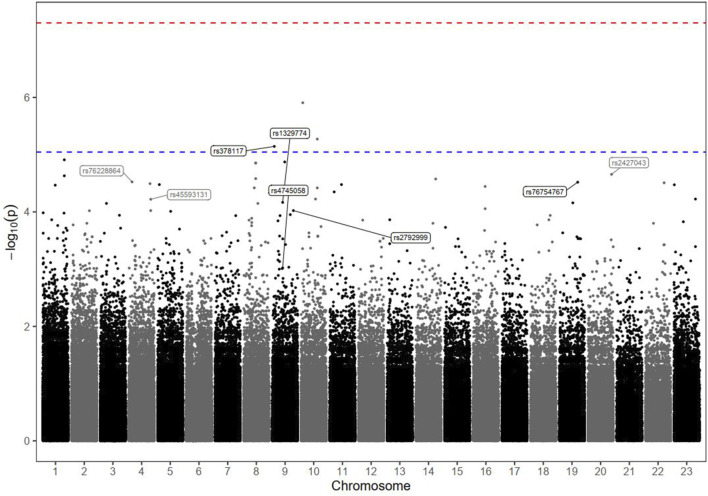
Manhattan plot of tumour progression markers in HCC patients receiving DOX chemotherapy. The plot shows the statistical association between each genomic variant and the outcome. The dots on the plot represent the chromosomal location on the x-axis and their statistical significance with respect to the phenotype of interest according to the logarithmic P value (-log10) on the y-axis. Each dot on the plot represents a single genomic variant. Variants that exceed the threshold line are statistically significant and are worth investigating. The–log10 P values of the detected SNPs were plotted across the 23 chromosomes. None of the SNPs met the genome-wide significance threshold (P < 5 × 10^−8^), as indicated by the red dashed line. Only the top markers of interest were annotated; the rs ID numbers of some SNPs above and below the blue dashed line (P < 9 × 10^−6^) are shown.

Table 2 and Figure 1 present the association results and Manhattan plots, respectively, of tumour progression outcomes in patients with advanced HCC receiving DOX. Table 2 shows the top six SNPs considered risk variants for enhanced progression of HCC tumours at P < 5 × 10^−5^. These SNPs were located within the AK3, TRPM3 (2 SNPs), CDH4, LINC00504, and GRIN2D loci. In contrast, two markers in two genes (HPGD and RC3H2) were demonstrated to protect against disease progression at P < 5 × 10^−5^.

**TABLE 2:** Top markers suggested as risk and protective factors for clinical symptomatic and radiological progression among HCC patients receiving DOX chemotherapy.

Variant	Allele	Chr	Gene	Gene function^a^	MAF %		P value	OR (95% CI)	Variant consequence
					Progressed cases^b^ (n = 35)	No progression (n = 43)			
Risk variants									
rs378117	G > A	9	AK3	Encodes the GTP:AMP and ITP:AMP phosphotransferase enzyme that regulates the metabolism of adenine nucleotides in the mitochondrial matrix	0.54	0.2	7.18E-06	4.8 (2.4–9.8)	Promoter
rs1329774	C > T	9	TRPM3 TRPM3	Encodes cation-selective channels that are important for cellular calcium signalling and homeostasis. These channels mediate calcium entry, affecting both cell death and proliferation	0.51	0.21	6.82E-05	4.0 (2.0–8.1)	Enhancer
rs4745058	C > T	9			0.43	0.19	0.000953	3.3 (1.6–6.7)	Enhancer
rs2427043	A > G	20	CDH4	Encodes the R-cadherins that are calcium-dependent cell adhesion proteins. They interact with themselves in a homophilic manner in connecting cells	0.36	0.08	2.21E-05	6.3 (2.5–15.7)	Enhancer
rs76228864	C > T	4	LINC00504	A long noncoding RNA that promotes cell proliferation, migration, and invasion	0.19	0.01	2.99E-05	19.4 (2.5–152)	Enhancer
rs76754767	G > A	19	GRIN2D	Encodes synaptic NMDA receptors with antiapoptotic activity, whereas the stimulation of extrasynaptic receptors caused a loss of the mitochondrial membrane potential and cell death	0.24	0.02	3.03E-05	13.5 (3.0–60.7)	TF binding site
Protective variant									
rs45593131	G > A	4	HPGD	Catalyses the NAD-dependent oxidation of prostaglandins, lipoxins and resolvins. This enzyme decreases the levels of the pro-proliferative prostaglandins such as prostaglandin E2 (whose activity is increased in cancer) and generates oxo-fatty acid products that can profoundly influence cell function by abolishing pro-inflammatory cytokine expression	0.04	0.29	6.02E-05	9.2 (2.6–31.8)	NMD
rs2792999	T > C	9	RC3H2	Encodes an RNA-binding protein involved in regulating gene expression at the posttranscriptional level. It can influence various cellular processes, including proliferation and invasion, which are critical in cancer progression	0.1	0.37	9.48E-05	5.3 (2.2–13.1)	NMD

^a^ Gene function data were derived from GeneCards (the human gene database, https://www.genecards.org/)

^b^ progression-free survival events (PFSEs) were defined objectively and subjectively based on two separate measures: a radiological examination (by imaging tests) or a clear clinical ascertainment of a symptomatic deterioration status.

Abbreviations: CI, confidence interval; MAF, minor allele frequency; NMD, nonsense-mediated decay; OR, odds ratio; TF, transcription factor.

The results of the Kaplan–Meier analysis, which confirmed the long-term significance of the effects of these identified top hit genotypes on PFS (during the study follow-up period), are described in Supplementary Figure S2. Interestingly, the estimated median PFS time was significantly shorter among HCC patients carrying the risk variant genotypes (in homozygous or heterozygous forms) than among those characterized by the reference status (log-rank P < 0.001). In contrast, the duration of the PFS period tended to be much longer among patients carrying protective variants than among those with major-allele genotypes (log-rank P < 0.001). The variants in linkage disequilibrium (LD) with the identified markers (r^2^ ≥ 0.6) are shown in Supplementary Excel Sheet 1. In addition, the haplotype analysis revealed no significant associations with any of the commonly detected haplotypes (Supplementary Excel Sheet 2).

### 3.4 Genetic associations with the biological response

**FIGURE 2:**
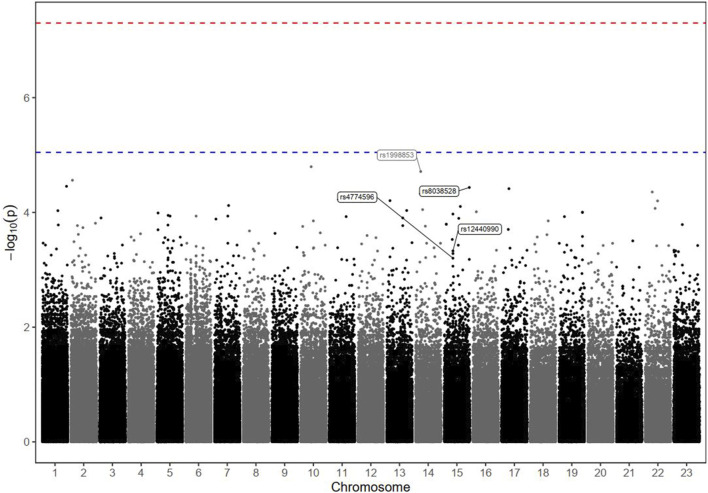
Manhattan plot of markers associated with the biological response (defined by the percentage of AFP reduction) in HCC patients receiving DOX chemotherapy. The–log10 P values of the detected SNPs were plotted across the 23 chromosomes. None of the SNPs met the genome-wide significance threshold (P < 5 × 10^−8^), as indicated by the red dashed line. Only four markers of interest were annotated; their rs ID numbers are shown below the blue dashed line (P < 9 × 10^−6^).

The GWAS analysis results for the tumour biological response in patients with advanced HCC receiving DOX are depicted in Table 3, and Manhattan plots are shown in Figure 2.

**TABLE 3:** Top markers associated with the AFP response among HCC patients receiving DOX chemotherapy.

Variant	Allele	Chromosome	Gene	Gene function^a^	MAF %		P value	OR (95% CI)	Variant consequence
					Good response^b^ (n = 34)	No response (n = 41)			
Low response									
rs8038528	A > G	15	PCSK6	Encodes PACE4, a calcium-dependent serine proteinase that participates in posttranslational modifications. PACE4 controls cell‒cell junctions by processing E-cadherin during blastocyst development	0.18	0.50	6.82E-05	4.0 (2.0–8.1)	NMD
High response									
rs1998853	A > G	14	NPAS3	A member of the neuronal PAS transcription factor gene family that has a variety of functions, including cancer development and neurobehaviour	0.41	0.11	1.94E-05	5.7 (2.4–13.2)	NMD
rs12440990	G > A	15	DMXL2	Encodes a transmembrane protein that is overexpressed in resistant breast cancer cell lines and activates the epithelial-to-mesenchymal transition	0.54	0.26	0.0004697	3.4 (1.7–6.7)	Enhancer
rs4774596	C > A	15	DMXL2		0.53	0.26	0.0006285	3.3 (1.6–6.6)	Promoter

^a^ Gene function data were derived from GeneCards (the human gene database, https://www.genecards.org/).

^b^ A greater than 20% reduction in the AFP level after TACE was considered a satisfactory endpoint for the tumour response.

Abbreviations: CI, confidence interval; MAF, minor allele frequency; NMD, nonsense-mediated decay; OR, odds ratio; TF, transcription factor.

Among the screened SNPs, only one polymorphism (rs8038528 in the PCSK6 gene) demonstrated the potential to be associated with a lower AFP response, where patients harbouring the variant (allele A) were (4 times) less likely to respond to DOX treatment based on an assessment of AFP% reduction levels (P = 6.82 × 10^−5^). In contrast, 3 SNPs (rs1998853, rs12440990, and rs4774596) located within 2 genes (NPAS3 and DMXL2) were suggested as high-response variants, where carriers of the variant alleles exhibited a greater probability of a biological AFP response (OR = 5.7, 3.4, and 3.3, respectively), however, at variable significance levels (Table 3).

LD data for the identified markers with r^2^ ≥ 0.6 are shown in Supplementary Excel Sheet 1. A haplotype analysis (Supplementary Excel Sheet 2) including the four mentioned SNPs revealed a significant association with the protective haplotype GA AA GG CC (p = 0.019).

### 3.5 Genetic associations with death and OS

**FIGURE 3:**
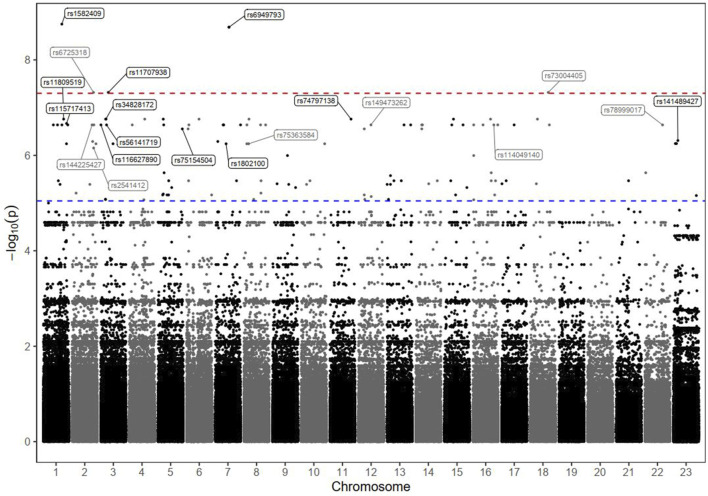
Manhattan plot of death markers in HCC patients receiving DOX chemotherapy. The–log10 P values of the detected SNPs were plotted across the 23 chromosomes. Five SNPs that met the genome-wide significance threshold (P < 5 × 10^−8^) are shown above the red dashed line. Other markers of interest were also annotated; their rs ID numbers are shown above the blue dashed line (P < 9 × 10^−6^).

The results of the GWAS analysis for the death incidence in patients with advanced HCC receiving DOX chemotherapy are displayed in Table 4, and the Manhattan plots are shown in Figure 3. As shown in Table 4, 20 markers of different genes had the potential to impact the incidence of death in our HCC cohort. Interestingly, 5 SNPs reached the genome-wide significance level (P < 5.0 × 10^−8^); 4 of them were located within defined genes (DENND1B, LOC107986086, TMEM169, and RNF152 loci), whereas one SNP, located on chromosome 7, was intergenic. The strongest observed signal was for the rs1582409 variant in the DENND1B (DENN domain containing 1B) locus on chromosome 1 (OR = 63.18, 95% CI = 6.77–589.4, P = 1.78 × 10^−9^).

**TABLE 4:** Important markers suggested as risk factors for death among HCC patients receiving DOX chemotherapy.

Variant	Chr	Allele	Gene	Gene function^a^	MAF %		P Value	OR (95% CI)	Variant consequence
					Death^b^ (n = 8)	Alive (n = 70)			
rs1582409	1	A > G	DENND1B	Controls cytokine production in TH2 lymphocytes by controlling the rate of T-cell receptor internalization and routing to endosomes	0.31	0.01	1.78E-09	63.18 (6.77–589.4)	NMD
rs6949793	7	C > T	–		0.25	0	2.06E-09	46.33 (4.79–448.17)	Enhancer
rs11707938	3	T > C	LOC107986086	An RNA gene, its specific biological role has not been clearly defined	0.31	0.01	4.80E-08	31.36 (5.45–180.7)	Regulatory
rs6725318	2	G > A	TMEM169	Encodes a protein that acts as a channel for the transport of certain chemicals across biological membranes	0.31	0.01	4.80E-08	31.36 (5.45–180.7)	Intronic
rs73004405	18	A > C	RNF152	Encodes a membrane-associated RING finger ubiquitin ligase that is thought to play a role in lysosome-related apoptosis	0.31	0.01	4.80E-08	31.36 (5.45–180.7)	Enhancer
rs11809519	1	A > C	KCNH1	Involved in the regulation of cell proliferation and differentiation, in particular the adipogenic and osteogenic differentiation of bone marrow-derived mesenchymal stem cells	0.25	0.01	1.74E-07	46.33 (4.79–448.2)	NMD
rs34828172	3	G > A	RBMS3	Encodes a protein that is implicated in cell cycle progression and apoptosis. It is activated in response to the presence of a tumour cell and acts to protect the cell	0.25	0.01	1.74E-07	46.33 (4.79–448.2)	NMD
rs74797138	11	A > G	GRIK4/LOC105369532	Encodes an ionotropic glutamate receptor that functions as a cation-permeable ligand-gated ion channel. It acts as the major excitatory neurotransmitter in the central nervous system	0.25	0.01	1.74E-07	46.33 (4.79–448.2)	Regulatory
rs115717413	1	C > T	GNG4	Predicted to enable G-protein beta-subunit binding activity and is involved in the negative regulation of cell growth	0.25	0.01	2.15E-07	45.67 (4.72–441.7)	Regulatory
rs114049140	16	G > T	PLCG2	Encodes a transmembrane signalling enzyme that catalyses the conversion of IP3 and DAG using calcium as a cofactor. Both IP3 and DAG are second messenger molecules that are important for transmitting signals from growth factor receptors and immune system receptors across the cell membrane	0.19	0	2.30E-07	32.08 (3.11–330.8)	NMD
rs116627890	3	C > T	CNTN6	Encodes a neuronal membrane protein that functions as a cell adhesion molecule. It may play a role in the formation of synapses in the developing nervous system	0.19	0	2.30E-07	32.08 (3.11–330.8)	NMD
rs144225427	2	C > T	BZW1-AS1	Translation initiation regulator	0.19	0	2.30E-07	32.08 (3.11–330.8)	Enhancer
rs149473262	12	ACAG/-	RAB3IP	Enables guanyl-nucleotide exchange factor activity and identical protein binding activity. Involved in several processes, including the positive regulation of cilium assembly and cell membranes	0.19	0	2.30E-07	32.08 (3.11–330.8)	NMD
rs56141719	3	C > A	TRIM71	Encodes an E3 ubiquitin-protein ligase that binds miRNAs and maintains the growth and upkeep of embryonic stem cells. Also involved in the G1-S phase transition of the cell cycle	0.19	0	2.30E-07	32.08 (3.11–330.8)	Enhancer
rs78999017	22	T > C	SHISAL1	Encodes a transmembrane adaptor protein that potentially interacts with other proteins to modulate cellular signalling pathways	0.19	0	2.30E-07	32.08 (3.11–330.8)	Enhancer
rs75154504	5	G > A	EIF4E1B	Predicted to enable RNA 7-methylguanosine cap-binding activity and translation initiation factor activity	0.19	0	2.79E-07	32.08 (3.11–330.8)	Noncoding exonic
rs141489427	X	G > C	PHEX	Encodes a transmembrane endopeptidase that is thought to be involved in bone and dentin mineralization and renal phosphate reabsorption	0.36	0.01	4.90E-07	49.71 (4.87–507.2)	NMD
rs1802100	7	A > G	NIPSNAP2	Encodes a NipSnap family protein that may be involved in vesicular transport. It is localized to mitochondria and plays a role in oxidative phosphorylation	0.31	0.02	5.72E-07	20.76 (4.37–98.54)	Enhancer
rs75363584	8	T > C	MTMR7	Encodes a lipid phosphatase that specifically dephosphorylates the D-3 position of phosphatidylinositol 3-phosphate	0.31	0.02	5.72E-07	20.76 (4.37–98.54)	NMD
rs2541412	2	C > T	IGFBP-AS1	Encodes insulin-like growth factor (IGF)--binding proteins, prolongs the half-life of the IGFs, and either inhibits or stimulates the growth-promoting effects of the IGFs on cultured cells	0.31	0.02	7.01E-07	20.45 (4.31–97.12)	TF binding site

^a^ Gene function data were derived from GeneCards (the human gene database, https://www.genecards.org/).

^b^ The number of death events that occurred at any time after TACE, treatment (OSe) and during the median OS, time, which was measured from the date of TACE, until the date of death from any cause, were estimated. Patients who survived until the end of the follow-up period (31 December 2023) comprised the surviving cohort.

Abbreviations: CI, confidence interval; MAF, minor allele frequency; NMD, nonsense-mediated decay; OR, odds ratio; TF, transcription factor.

The results of the Kaplan–Meier analysis are shown in Supplementary Figure S3. The estimated median OS time tended to be much shorter among the patients carrying any of the identified risk variants (in homozygous or heterozygous forms) than among those characterized by the reference status (log-rank P < 0.001).

Furthermore, the LD data for the identified markers with r^2^ ≥ 0.6 are shown in Supplementary Excel Sheet 1. A 10 SNP haplotype analysis (including the top ten associated markers in Supplementary Excel Sheet 2) revealed a significant association with the protective haplotype AA CC TT GG AA AA GG AA GG GG CC (P = 0.00000103).

## 4 Discussion

This pharmacogenomic study used a GWAS approach to overcome the shortcomings of association findings obtained from previous studies that focused on candidate genes related to DOX therapy (Supplementary Table S1 **)** in various types of cancer, including HCC (Nyangwara et al., 2024; Ruiz-Pinto et al., 2018; Hertz et al., 2016; Lal et al., 2016; Faraji et al., 2016; Wojnowski et al., 2005; Windsor et al., 2011; Gelderblom et al., 2014; Gándara-Mireles et al., 2021; Liang et al., 2016). To our knowledge, this study is the first to examine the ability of a large portion of the genome to modulate the clinical response of HCC patients to DOX therapy. The associations discussed here are confined to the suggested top genetic variants linked to each clinical outcome (tumour progression, tumour response, and death).

Several genes have been suggested to modulate tumour progression and the biological response, although these genes did not reach the genome-wide association significance level of 5.0 × 10^−8^. In contrast, strong genetic associations were detected with a potential impact on the death outcome in this HCC cohort. Notably, none of the genes associated with death overlapped with the identified genes involved in tumour progression and reduced AFP levels. This finding likely indicates alternative molecular mechanisms underlying each outcome. Consequently, we attempted to interpret the observed associations in terms of the molecular mechanisms underlying gene–HCC and gene–DOX interactions in light of updated research and the scientific literature.

### 4.1 Progression-related variants in HCC

The AK3 gene encodes the adenylate kinase (AK) 3 protein, which is a GTP:AMP and ITP:AMP phosphotransferase enzyme found in the mitochondrial matrix (Liu et al., 2019). The SNP rs378117 in AK3 is a promoter variant associated with reduced gene activity and probably serves as a predictor of progressive symptoms and radiological deterioration of the tumour in our cohort. The literature revealed contradictory findings of the enhanced metastasis of cancer cells with over- or underexpression of genes encoding AK isoforms and, consequently, enzymatic changes depending on the tissue specificity and different phases (initial vs advanced stages of tumour growth) (Klepinin et al., 2020). Consistent with our observations, some studies have shown that AK3 is downregulated in lung cancer (Balinsky et al., 1984) and hepatoma (Criss et al., 1970). Similarly, an *in vitro* cell line study showed that AK3 knockout was associated with reduced cell proliferation and increased expression of oxidative stress-related proteins, indicating the importance of this enzyme in maintaining mitochondrial homeostasis (Fujisawa et al., 2022). Moreover, a subsequent study documented significantly lower levels of AK3 expression in thiopurine-resistant human leukaemia cells than in wild-type cells, indicating a role for the AK3 variant in metastasis progression (Karim et al., 2011). Additionally, the suppression of AK3 (as a phosphotransferase and glycolysis-related gene) triggers a cancerous transformation, leading to an uncontrolled cell cycle and growth with a subsequently worse prognosis and shorter survival of patients with breast cancer (Klepinin et al., 2020; Chao et al., 2024). Our study is the first to report the novel impact of AK3 (rs378117) on increasing the progression of human HCC.

The product of the TRPM3 gene belongs to the family of transient receptor potential (TRP) channels. TRP channels are cation-selective channels that are important for cellular calcium signalling and homeostasis. The protein encoded by this gene mediates calcium entry, consequently affecting both cell death and proliferation (Clapham et al., 2005; Gkika and Prevarskaya, 2009). A large-scale database analysis revealed that the level of the TRPM3 transcript was decreased in the majority of cancer tissues. Conversely, the Kaplan–Meier analysis revealed that high TRPM3 mRNA expression levels were significantly associated with an improved prognosis and increased OS of all examined patients with various cancer subtypes (Qin et al., 2020). Similarly, the variants detected within the TRPM3 gene (rs1329774 and rs4745058) in our study are likely linked to downregulated gene expression and thus are significantly associated with tumour progression. These results suggest that targeting ion channels such as TRPM3 to overcome drug resistance in cancer treatment could be an active area for drug discovery and development.

The CDH4 gene, which encodes the R-cadherin protein, has been significantly implicated in the progression and treatment of various cancers. However, the expression level and function of CDH4 in different types of cancer remain controversial (Aacr Project GENIE Consortium, 2017). In some cancers, it acts as an oncogene (bladder cancer), whereas in others, it acts as a tumour suppressor or antioncogene (such as in lung cancer, nasopharyngeal carcinoma, gastric cancer, and colorectal cancer) (Uhlen et al., 2017; Li et al., 2017). This dual role may complicate the development of therapies targeting CDH4 (Xie J.et al., 2023). The specific role and expression of CDH4 in HCC were not previously defined. Interestingly, our data revealed a CDH4 variant (rs2427043) with a potential impact on the progression of tumours in the HCC population, reflecting an increased risk of downregulated expression of the CDH4 protein. Similarly, a previous GWAS revealed that two other CDH4 variants (rs1122269 and rs4925193) were associated with significant trends (although not at the level of the current study) in modifying the OS outcomes of patients receiving gemcitabine treatment for pancreatic cancer (Li et al., 2016). Our results, together with those of gemcitabine studies, imply the importance of further functional and clinical studies to define the role of the CDH4 gene as a PGx biomarker for the individualization of chemotherapy or as a novel target for cancer drug development.

LINC00504 is a long noncoding RNA (lncRNA) that is associated with several types of cancer, including HCC. It is highly expressed in cancer cells compared with normal cells, where it acts as an oncogene associated with an unfavourable prognosis (Mathias et al., 2021; Hou et al., 2023). Its overexpression is associated with aggressive tumour characteristics and correlates with poorer patient outcomes. Elevated levels of LINC00504 correlate with an increased tumour size, advanced stage, and lymph node metastasis, indicating a negative impact on the prognosis (Zhang, 2020). On the other hand, LINC00504 has been shown to influence DOX resistance in HCC cells through various mechanisms, specifically by influencing apoptotic pathways, promoting the epithelial‒mesenchymal transition (EMT), and enhancing metabolic adaptations (Shan and Li, 2019). High levels of LINC00504 are associated with reduced sensitivity to DOX in HCC cells (Ma et al., 2019). Studies indicate that HCC cell lines with elevated LINC00504 expression tend to show greater resistance to DOX treatment than those with lower expression levels (Mathias et al., 2021). Our findings revealed a high allele frequency (19%) of rs76228864 in LINC00504 among patients with tumour progression versus only 1% in the control group. This finding reflects the strong association of the LINC00504 marker with the risk of HCC progression.

GRIN2D, which encodes the GluN2D subunit of N-methyl-D-aspartate receptors (NMDARs), has been identified as a potential oncogene and a novel therapeutic target in certain types of cancer (Wang et al., 2023). For example, in pancreatic and colorectal cancer, GRIN2D was overexpressed in patients’ tissues at both the mRNA and protein levels. Consequently, upregulated GRIN2D could effectively promote tumour growth and liver metastasis by activating subcellular signalling pathways and transcription factors. Furthermore, high expression levels of GRIN2D in HCC are associated with increased infiltration of various immune cells, including T cells and natural killer (NK) cells. This finding suggests that GRIN2D may facilitate a more active immune response within the tumour microenvironment, potentially impacting tumour progression and the patient prognosis (Zhang Y. G. et al., 2022; Xie Z. et al., 2023).

Additionally, high expression levels of GRIN2D are associated with increased resistance to DOX, potentially through its effects on the expression of P-glycoprotein (P-gp), a well-known efflux pump that contributes to multidrug resistance by actively transporting drugs out of cells, thereby reducing efficacy (Zhang Z. et al., 2022). Accordingly, combination therapies that incorporate DOX with agents targeting GRIN2D or related pathways are being explored as strategies to improve therapeutic outcomes in patients with HCC (Duan and Liu, 2018). Consistent with previous reports, our results identified a transcription factor variant in GRIN2D (rs76754767) as a risk factor for HCC tumour progression.

The HPGD gene is recognized as a tumour suppressor in various cancers, including HCC. Its function involves the degradation of prostaglandins, particularly PGE2, which is known to promote inflammation and tumour progression. The downregulation of HPGD is associated with increased levels of PGE2 in the tumour microenvironment, leading to the increased proliferation and survival of hepatocytes, which contributes to the development of HCC from metabolic dysfunction-associated steatohepatitis (Hu et al., 2023). Therefore, restoring HPGD levels or inhibiting PGE2 signalling has been explored as a therapeutic option to mitigate tumour growth and improve the immune response or apoptosis signalling pathways (Ren et al., 2023; Li C. J. et al., 2023). Interestingly, our data revealed a protective relationship between the HPGD variant rs45593131 and HCC progression. RC3H2 (an RNA-binding protein with multiple C3H-type zinc fingers) is involved in regulating gene expression at the posttranscriptional level. It can influence various cellular processes, including proliferation and invasion, which are critical in cancer progression (Zhang et al., 2015).

Studies have shown that RC3H2 is upregulated in HCC tissues compared with normal liver tissues. Its overexpression correlates with increased cell proliferation and invasion capabilities, suggesting that RC3H2 may play a significant role in the aggressiveness of HCC (Zhao et al., 2023).

Consistent with these reports, our data revealed that rs2792999 in RC3H2 is a protective variant with a strong negative association with HCC tumour progression. This variant is likely associated with decreased RC3H2 expression.

### 4.2 AFP response-related variants

The PCSK6 gene (proprotein convertase subtilisin/kexin type 6), which is located on chromosome 15q26.3, encodes PACE4, a calcium-dependent serine proteinase that participates in posttranslational modifications. PACE4 controls cell‒cell junctions by processing E-cadherin during blastocyst development. PCSK6 is upregulated in pancreatic cancer liver metastases, and its inactivation results in the decreased migratory potential of tumour cells in pancreatic cancer and reprogramming of cell‒cell junctions (He et al., 2022). However, in the liver, PCSK9 binds to and degrades the low-density lipoprotein receptor, which is a major contributor to hypercholesterolemia. Therefore, PCSK6 plays an important role in the cardiovascular system and homeostasis (Suur et al., 2021; Bordicchia et al., 2019). A previous study (using an animal model) showed that the overexpression of PCSK6 protects the heart from the long-term damage caused by DOX, reduces cardiac oxidative stress, decreases apoptosis, restores the autophagolysosomal degradation process that is inhibited by DOX, and increases autophagy (Li C. et al., 2023).

In addition, studies on PCSK6 expression in cell lines revealed a PCSK6 variant (rs7181043) that was significantly associated with the plaque fat content in atherosclerotic patients (Suur et al., 2021; Bordicchia et al., 2019). PCSK6 variants are predicted to decrease gene function, which may increase DOX-induced cardiotoxicity. Consistently, our study reported that rs8038528 in the PCSK6 gene, a low-response variant, was associated with a lower AFP% reduction after DOX therapy. Based on the available findings, the PCSK6 expression level appears to be related to the AFP% change in HCC patients and is potentially related to DOX treatment.

We also identified the rs12440990 and rs4774596 SNPs, which are located within the DMXL2 gene, as high-response variants. Carriage of these variants is likely linked to an AFP% reduction. DMXL2 is a transmembrane protein that is overexpressed in resistant breast cancer cell lines, is an activator of the epithelial-to-mesenchymal transition, and is a potential new biomarker for hormone-positive breast cancer (Valter et al., 2023; Faronato et al., 2015). Nevertheless, the exact mechanisms of DMXL2 involvement in HCC are still being investigated (Ikeda et al., 2014).

The marker rs1998853, located in NPAS3 on chromosome 14, also showed a greater response, with an AFP% reduction following DOX treatment. NPAS3 is a member of the neuronal PAS transcription factor gene family, which has a variety of functions, including cancer development and neurobehaviour. NPAS3, a transcription factor, has a tumour-suppressive role in manipulating the progression of certain types of cancer (Moreira et al., 2011; Yu et al., 2024). The absence of NPAS3 expression in glioblastomas (a type of brain cancer) was reported to be associated with shorter OS. Overexpression of NPAS3 in malignant glioma cell lines dramatically slowed transformation, whereas lower expression spurred more aggressive development (Moreira et al., 2011). Although NPAS3 was historically connected to neurogenesis, it has emerged as a predictive hallmark for triple-negative breast cancer as a tumour suppressor that drives the progression of breast cancer via the modulation of autophagy (Yu et al., 2024).

### 4.3 Death-related variants in HCC

The highest detected death signal (P = 1.78 × 10^−09^) was for the rs1582409 variant located in the DENND1B (DENN domain containing 1B) locus on chromosome 1. Previously, DENND1B was associated with autoimmune liver disease in genome-wide meta-analyses (Huang et al., 2023; Liu et al., 2010). It has also been implicated in certain types of cancer. For example, a circular RNA form of DENND1B (circ_DENND1B) was found to inhibit tumorigenicity in clear cell renal cell carcinoma (ccRCC) (Chen et al., 2022). These findings suggest that DENND1B may have tumour-suppressive properties in certain contexts. Furthermore, a few SNPs in DENND1B have been reported to increase the risks of pancreatic, renal and gastric cancers (Cotterchio et al., 2015; Nookala et al., 2012).

DOX can cause significant side effects, including cardiotoxicity (Nyangwara et al., 2024; Hertz et al., 2016; Wojnowski et al., 2005; Gándara-Mireles et al., 2021). The rs1582409 variant in the DENND1B gene might influence the likelihood or severity of cardiotoxicity leading to death.

The SNP rs6725318, which is located in the TMEM169 gene on chromosome 2, may play a role as a risk factor for death in HCC patients receiving DOX therapy. TMEM is a protein that functions as a channel for the transport of certain chemicals across biological membranes. TMEM9 was found to be abnormally expressed in liver cancers (Zhang et al., 2016), and it was described as an oncogene implicated in tumour development, invasion, and chemoresistance (Schmit and Michiels, 2018). A TMEM169 gene mutation (rs6725318) is predicted to contribute to DOX resistance by lowering transmembrane protein activity, possibly altering drug uptake, efflux, and accumulation in cells. These properties may partially explain the suppression of the apoptosis pathway, which is a mechanism of DOX resistance.

The present study also revealed a strong association between rs73004405 in RNF152 on chromosome 18 and the death incidence. RNF152, a membrane-associated RING finger ubiquitin ligase, is found in lysosomes and is thought to play a role in lysosome-related apoptosis, resulting in the inhibition of HCC cell growth (Ueda et al., 2024).

Furthermore, RING finger-related E3 ubiquitin ligases play a role in carcinogenesis and can act as oncogenes or tumour suppressors, depending on the target proteins. RNF152 was previously reported to be highly downregulated in HCC and associated with decreased OS and PFS (Wan et al., 2021). Similarly, the expression of RNF152 in colorectal cancer (CRC) tissues is considerably lower than its expression in adjacent noncancerous tissues. In patients with CRC, high expression levels of RNF152 are associated with a better prognosis, whereas low expression of RNF152 is associated with lymphatic metastases (Cui et al., 2018). RNF152 is known to play a role in regulating apoptosis and autophagy (Ueda et al., 2024).

Another RNF gene (RNF6) was also identified by Cai et al. (Cai et al., 2019) as an independent predictor of poor outcomes in patients with HCC. In a previous HCC study, RNF6 silencing inhibited radioresistance and the EMT both *in vivo* and *in vitro*. Forkhead box protein A1 (FoxA1), a crucial transcriptional repressor of the EMT, is directly bound and ubiquitylated by RNF6, suggesting that FoxA1 degradation is partially responsible for the carcinogenic effect of RNF6 on HCC (Liu et al., 2010). Overall, these results are consistent with our findings, which indicate an important association between the rs73004405 variant in RNF152 and the survival of HCC patients treated with DOX. Given the effects of DOX on apoptotic pathways, some interplay may exist between the drug’s action and RNF152’s function in regulating cell death.

### 4.4 Summary outcomes

The genes explored in our study are novel and different from those previously reported. As illustrated in Supplementary Table S1, most of the previously reported genes are known to influence DOX PKs (e.g., distribution (transport) and metabolism) rather than DOX PDs (e.g., efficacy and toxicity) (Gonzalez-Covarrubias et al., 2009; Mordente et al., 2003; Nyangwara et al., 2024; Ruiz-Pinto et al., 2018) in heterogeneous types of cancer (Hertz et al., 2016; Lal et al., 2016; Faraji et al., 2016; Wojnowski et al., 2005; Windsor et al., 2011). These genes include SLC transporters, which mediate DOX uptake into cancer cells [e.g., SLC22A16 (organic cation transporter)]; ABC transporters, which are involved in the efflux of DOX and contribute to drug resistance [e.g., ABCB1 (MDR1), ABCC1 (MRP1), and ABCG2 (BCRP)]; RABLP, which may influence the intracellular trafficking of DOX; and CBR enzymes [e.g., CBR1 and CBR3] or AKR1A, which are involved in DOX metabolism and detoxification. In contrast, our GWAS explored various genes (AK3, TRPM3, CDH4, LINC00504, GRIN2D, HPGD, RC3H2, PCSK6, NPAS3, and DMXL2), which had significantly variable impacts on clinical outcomes of DOX after TACE in HCC patients. These genes are suggested to be involved in diverse DOX PD-related biological processes, such as nucleotide metabolism (AK3), calcium signalling (TRPM3 and GRIN2D), cell adhesion and invasion (CDH4 and DMXL2), posttranscriptional and posttranslational regulation (RC3H2 and PCSK6), transcription regulation (NPAS3) and prostaglandin metabolism (HPGD).

### 4.5 Limitations

First, the associations obtained in this study are limited to the tested Arabic cohort and may not be generalizable unless they are replicated in diverse populations. While evidence of a shared genetic architecture for many traits has been reported, significant differences exist in risk allele frequencies and effect sizes between populations of different ethnicities (Chen et al., 2022). Second, GWAS findings are highly replicable, with some studies showing replication rates of up to 94% for markers that meet the GWAS cut-off level (P ≤ 5.0 × 10^−8^) (Ntzani et al., 2011). The reported associations with the PFS and the AFP response of patients with HCC did not reach the genome-wide significance threshold; thus, replication in another independent study is recommended. Third, although the detected risk-of-death markers met the GWAS significance level, the analysis of a small number of death incidents (n = 8) may lack sufficient statistical power to detect true associations. Hence, further replication studies may be necessary to confirm our findings. Fourth, genotype-based association tests were not performed as substantially larger sample sizes would typically be required to achieve a satisfactory power. This can be more resource-intensive and challenging to implement in the current study. Finally, this study report focuses on genetic factors influencing specific disease outcomes, such as the DOX response and survival, to analyze their roles in inter-individual variability. It identifies key genetic variants and their functional implications, uncovering the potential biomarkers and pathways contributing to treatment outcomes. However, non-genetic factors such as environmental exposures, lifestyle, and comorbidities may also play a significant role in shaping disease outcomes. Future larger sample studies could allow us to integrate findings from both genetic and non-genetic analyses for more effective risk prediction, treatment optimization, and personalized patient care.

## 5 Conclusion

In summary, this study identified novel genetic variants that may play significant roles in mediating DOX resistance and variants linked with a good prognosis in a real-world cohort of HCC patients. However, further replication in larger multiethnic studies remains mandatory before their implementation in precision medicine practice. Additionally, the findings suggest that biochemical investigations targeting these variants could lead to the development of different treatment options to overcome the resistance and limited efficacy of DOX-based therapies for HCC patients. Further research is necessary to fully elucidate these mechanisms and explore therapeutic strategies that could mitigate the impacts of these markers on drug resistance, PFS, and/or OS of patients with HCC.

## Data Availability

The original contributions presented in the study are included in the article/Supplementary Material, further inquiries can be directed to the corresponding author.
