# Supplementary material for: Genetic polymorphisms as predictors of the response of hepatocellular carcinoma patients to doxorubicin chemotherapy: a genome-wide association study
Source: Front Pharmacol. 2025 Jun 4;16:1604473. doi: 10.3389/fphar.2025.1604473 (PMC12174396; doi:10.3389/fphar.2025.1604473)
Supplement: Supplementary file 1 [file Table1.docx]

**Supplementary Table 1: List of known genetic markers identified in literature versus those explored in the present study.**

| **Category** | **Previously Reported Genes** | **Genes Explored in This Study** | **Overlap/Difference** |
| --- | --- | --- | --- |
| **Drug Metabolism (PK)** | ***CBR1, CBR3 ^1^*** |  |  |
|  | ***AKR1A ^2^***  (aldo-keto reductases (AKRs), which convert doxorubicin to doxorubicinol) |  |  |
|  | ***NOS3 ^3,4^***  DOX induce NOS expression and produce elevated ROS, |  |  |
|  | ***UGT1A6 ^4^***  detoxification glucuronidation pathway and reduce Dox –clearance |  |  |
| **Drug Transport (PK)** | ***ABCB1 (MDR1), ABCC1 (MRP1) ^4^***  Dox resistance and poor survival | ***DMXL2 ^5^*** | Overlap: Both identify genes linked to drug resistance (efflux vs. Epithelial mesenchymal transition (EMT) |
|  | ***SLC22A16, SLC28A3 ^6,7^***  doxorubicin uptake |  |  |
| **Oxidative stress** | ***SOD2*** ***^8^*** |  |  |
|  | ***GSTP1 ^4^***  encodes glutathione S-transferase M1, that detoxify DOX anthracycline |  |  |
|  | ***HFE ^9^***  hemochromatosis (HFE) gene mutations doxorubicin-associated cardiotoxicity |  |  |
|  | ***RAC2 ^3^***  NAD(P)H oxidase complex (CYBA, NCF4, and RAC2), involved in the generation of reactive oxygen species (ROS) or reactive nitrogen species (RNS) from doxorubicin metabolism |  |  |
| **Drug dynamics** | ***TOP2A* ^3^** doxorubicin resistance & DNA repair mechanisms and the cell cycle control | **AK3 ^10^**  Adenylate kinase (AK) | Difference: Novel gene regulates adenine nucleotide metabolism, maintaining intracellular nucleotide metabolic homeostasis & Interfere with apoptosis signalling pathways |
|  |  | ***RNF152 ^11^***  ubiquitin ligase | Difference: Novel gene induce apoptosis |
|  |  | ***PCSK6 ^12^*** | Difference: Novel gene focuses on the maintenance of cardiac function and vascular homeostasis. |
| **Oxidative Stress** | ***CYBA ^8^*** |  |  |
|  | ***RARG ^13^***  retinoic acid receptor-γ increased risk of anthracycline-induced cardiotoxicity. is mediated via topoisomerase 2β (TOP2B) |  |  |
| **Cell Adhesion/Invasion** |  | **RC3H2 ^14,15^** | Difference: Novel gene focuses on cardiovascular disease risk, cell growth, formation, migration, invasion |
|  |  | ***CDH4 ^16,17^*** | Difference: Novel genes focus on cell adhesion and invasion and promote apoptosis |
|  |  | ***LINC00504 ^18,19^*** | Difference: Novel genes focus on cell adhesion and invasion and promote apoptosis |
| **Calcium Signaling** |  | ***TRPM3, ^20,21^*** | Difference: Novel gene focuses on tumor epithelial-mesenchymal transition (EMT), autophagy, and cancer metabolic reprogramming |
|  |  | ***GRIN2D ^22.23^*** | Difference: Novel genes focus on calcium signaling pathways. |
| **Inflammatory Response** | **IL-6 ^24^/IL-10 ^25^** | **HPGD ^26,27^** | Difference: Novel gene focuses on metabolism of prostaglandins, which participate in inflammation & angiogenesis and |
|  |  | **DENND1B ^28^** | Difference: Novel genes regulate cell inflammation and apoptosis. |
| **Transcription Regulation** |  | **NPAS3 ^29^** | Difference: Novel gene focuses on proliferation and apoptosis |

**References**

1. Lal S, Sandanaraj E, Wong ZW, et al. CBR1 and CBR3 pharmacogenetics and their influence on doxorubicin disposition in Asian breast cancer patients. *Cancer Sci*. 2008;99(10):2045-2054. doi:10.1111/j.1349-7006.2008.00903.x
2. Heibein AD, Guo B, Sprowl JA, Maclean DA, Parissenti AM. Role of aldo-keto reductases and other doxorubicin pharmacokinetic genes in doxorubicin resistance, DNA binding, and subcellular localization. *BMC Cancer*. 2012;12:381. Published 2012 Aug 31. doi:10.1186/1471-2407-12-381
3. Thorn CF, Oshiro C, Marsh S, et al. Doxorubicin pathways: pharmacodynamics and adverse effects. *Pharmacogenet Genomics*. 2011;21(7):440-446. doi:10.1097/FPC.0b013e32833ffb56
4. Ding Y, Du K, Niu YJ, Wang Y, Xu X. Genetic Susceptibility and Mechanisms Underlying the Pathogenesis of Anthracycline-Associated Cardiotoxicity. *Oxid Med Cell Longev*. 2022;2022:5818612. Published 2022 Aug 3. doi:10.1155/2022/5818612
5. Valter A, Luhari L, Pisarev H, et al. Genomic alterations as independent prognostic factors to predict the type of lung cancer recurrence. *Gene*. 2023;885:147690. doi:10.1016/j.gene.2023.147690
6. Bagdasaryan AA, Chubarev VN, Smolyarchuk EA, et al. Pharmacogenetics of Drug Metabolism: The Role of Gene Polymorphism in the Regulation of Doxorubicin Safety and Efficacy. *Cancers (Basel)*. 2022;14(21):5436. Published 2022 Nov 4. doi:10.3390/cancers14215436
7. Fonoudi H, Jouni M, Cejas RB, et al. Functional Validation of Doxorubicin-Induced Cardiotoxicity-Related Genes. *JACC CardioOncol*. 2024;6(1):38-50. Published 2024 Jan 23. doi:10.1016/j.jaccao.2023.11.008
8. Miriyala, S., et al. MnSOD in oxidative stress signaling and cancer: mechanisms and therapeutic opportunities. Antioxidants & Redox Signaling, 2021; 35(6), 487-509. https://doi.org/10.1089/ars.2020.8169
9. Lipshultz SE, Lipsitz SR, Kutok JL, et al. Impact of hemochromatosis gene mutations on cardiac status in doxorubicin-treated survivors of childhood high-risk leukemia. *Cancer*. 2013;119(19):3555-3562. doi:10.1002/cncr.28256
10. Fujisawa K, Wakazaki M, Matsuzaki A, et al. Adenylate Kinase Isozyme 3 Regulates Mitochondrial Energy Metabolism and Knockout Alters HeLa Cell Metabolism. *Int J Mol Sci*. 2022;23(8):4316. Published 2022 Apr 13. doi:10.3390/ijms23084316
11. Okamoto T, Imaizumi K, Kaneko M. The Role of Tissue-Specific Ubiquitin Ligases, RNF183, RNF186, RNF182 and RNF152, in Disease and Biological Function. *Int J Mol Sci*. 2020;21(11):3921. Published 2020 May 30. doi:10.3390/ijms21113921
12. Zhan W, Chen L, Liu H, et al. *Pcsk6* Deficiency Promotes Cardiomyocyte Senescence by Modulating Ddit3-Mediated ER Stress. *Genes (Basel)*. 2022;13(4):711. Published 2022 Apr 18. doi:10.3390/genes13040711
13. Magdy T, Jiang Z, Jouni M, et al. RARG variant predictive of doxorubicin-induced cardiotoxicity identifies a cardioprotective therapy. *Cell Stem Cell*. 2021;28(12):2076-2089.e7. doi:10.1016/j.stem.2021.08.006
14. Hahn J, Fu YP, Brown MR, et al. Genetic loci associated with prevalent and incident myocardial infarction and coronary heart disease in the Cohorts for Heart and Aging Research in Genomic Epidemiology (CHARGE) Consortium. *PLoS One*. 2020;15(11):e0230035. Published 2020 Nov 13. doi:10.1371/journal.pone.0230035
15. Wu K, Jiang Y, Zhou W, et al. Long Noncoding RNA RC3H2 Facilitates Cell Proliferation and Invasion by Targeting MicroRNA-101-3p/EZH2 Axis in OSCC. *Mol Ther Nucleic Acids*. 2020;20:97-110. doi:10.1016/j.omtn.2020.02.006
16. Zhang N, Li L, Long Z, et al. Are dietary factors involved in the association of *CDH4* methylation and breast cancer risk?. *Br J Nutr*. 2022;127(12):1868-1877. doi:10.1017/S0007114521002804
17. Li L, Zhang JW, Jenkins G, et al. Genetic variations associated with gemcitabine treatment outcome in pancreatic cancer. *Pharmacogenet Genomics*. 2016;26(12):527-537. doi:10.1097/FPC.0000000000000241
18. Hou T, Ye L, Wu S. Knockdown of LINC00504 Inhibits the Proliferation and Invasion of Breast Cancer via the Downregulation of miR-140-5p. *Onco Targets Ther*. 2021;14:3991-4003. Published 2021 Jul 2. doi:10.2147/OTT.S294965
19. Zhang Z. Silencing LINC00504 inhibits cell proliferation, invasion as well as migration and promotes cell apoptosis in lung cancer cells via upregulating miR-876-3p. *Cytotechnology*. Published online October 8, 2020. doi:10.1007/s10616-020-00424-5
20. Ciaglia T, Vestuto V, Bertamino A, González-Muñiz R, Gómez-Monterrey I. On the modulation of TRPM channels: Current perspectives and anticancer therapeutic implications. *Front Oncol*. 2023;12:1065935. Published 2023 Feb 9. doi:10.3389/fonc.2022.1065935
21. Oberwinkler J, Lis A, Giehl KM, Flockerzi V, Philipp SE. Alternative splicing switches the divalent cation selectivity of TRPM3 channels. *J Biol Chem*. 2005;280(23):22540-22548. doi:10.1074/jbc.M503092200
22. Wang J, Wong CH, Zhu Y, et al. Identification of GRIN2D as a novel therapeutic target in pancreatic ductal adenocarcinoma. *Biomark Res*. 2023;11(1):74. Published 2023 Aug 8. doi:10.1186/s40364-023-00514-4
23. Wang LL, Li J, Xue H, et al. The promoting effects of Grin2d expression in tumorigenesis and the aggressiveness of esophageal cancer. *Histol Histopathol*. 2024;39(5):659-670. doi:10.14670/HH-18-674
24. Kishimoto T. IL-6: from its discovery to clinical applications. *Int Immunol*. 2010;22(5):347-352. doi:10.1093/intimm/dxq030
25. Moore, K. W., et al. Interleukin-10 and the interleukin-10 receptor: signaling and regulation in immune responses." Annual Review of Immunology, 2020; 38, 1-24. <https://doi.org/10.1146/annurev-immunol-042718-041718>
26. Qi X, Wang Y, Hou J, Huang Y. A Single Nucleotide Polymorphism in *HPGD* Gene Is Associated with Prostate Cancer Risk. *J Cancer*. 2017;8(19):4083-4086. Published 2017 Oct 24. doi:10.7150/jca.22025
27. Beccacece L, Abondio P, Bini C, Pelotti S, Luiselli D. The Link between Prostanoids and Cardiovascular Diseases. *Int J Mol Sci*. 2023;24(4):4193. Published 2023 Feb 20. doi:10.3390/ijms24044193
28. Chen Y, Wang Z, Chen X, Peng X, Nie Q. CircNFIC Balances Inflammation and Apoptosis by Sponging miR-30e-3p and Regulating DENND1B Expression. *Genes (Basel)*. 2021;12(11):1829. Published 2021 Nov 19. doi:10.3390/genes12111829
29. Luoma LM, Berry FB. Molecular analysis of NPAS3 functional domains and variants. *BMC Mol Biol*. 2018;19(1):14. Published 2018 Dec 3. doi:10.1186/s12867-018-0117-4
