# Supplementary material for: Genetic polymorphisms as predictors of the response of hepatocellular carcinoma patients to doxorubicin chemotherapy: a genome-wide association study
Source: Front Pharmacol. 2025 Jun 4;16:1604473. doi: 10.3389/fphar.2025.1604473 (PMC12174396; doi:10.3389/fphar.2025.1604473)
Supplement: Supplementary file 3 [file Image2.pdf]

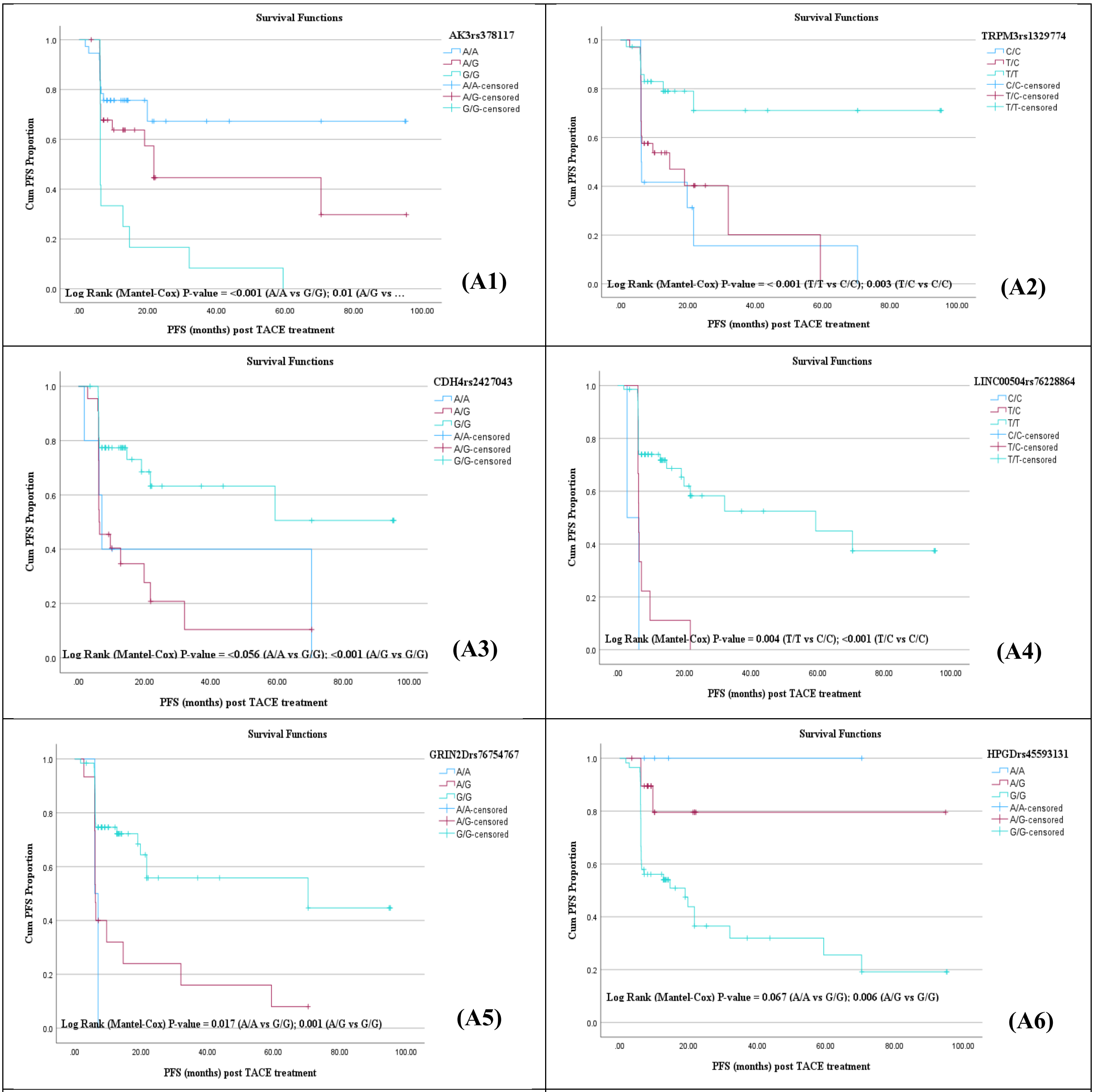

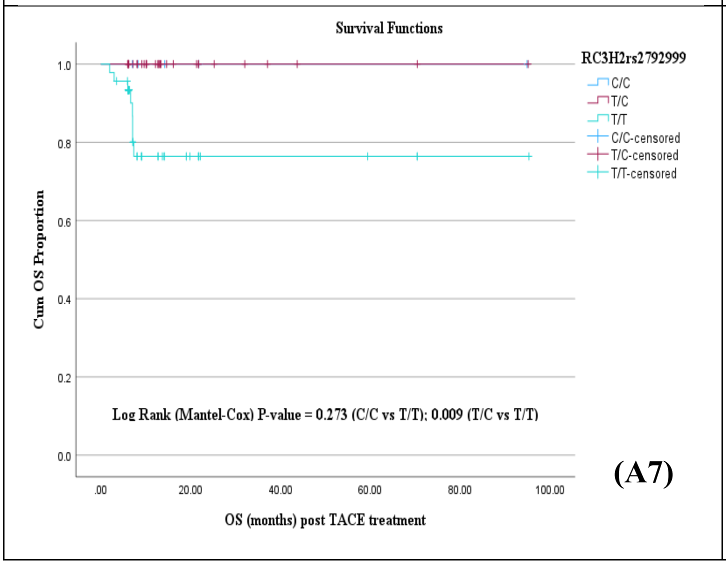

Supplementary Figure 2: Kaplan–Meier analysis of progression-free survival (PFS) in HCC patients receiving doxorubicin chemotherapy mediated through TACE. (A1) PFS in patients with different AK3 rs378117 genotypes. **(A2)** PFS in patients with different TRPM3 rs1329774 genotypes. **(A3)** PFS in patients with different CDH4 rs2427043 genotypes. **(A4)** PFS in patients with LINC00504 rs76228864 different genotypes. **(A5)** PFS in patients with different GRIN2D rs76754767 genotypes. **(A6)** PFS in patients with different HPGD rs45593131 genotypes. **(A7)** PFS in patients with different RC3H2 rs2792999 genotypes.
