# Supplementary material for: Genetic polymorphisms as predictors of the response of hepatocellular carcinoma patients to doxorubicin chemotherapy: a genome-wide association study
Source: Front Pharmacol. 2025 Jun 4;16:1604473. doi: 10.3389/fphar.2025.1604473 (PMC12174396; doi:10.3389/fphar.2025.1604473)
Supplement: Supplementary file 5 [file Image3.pdf]

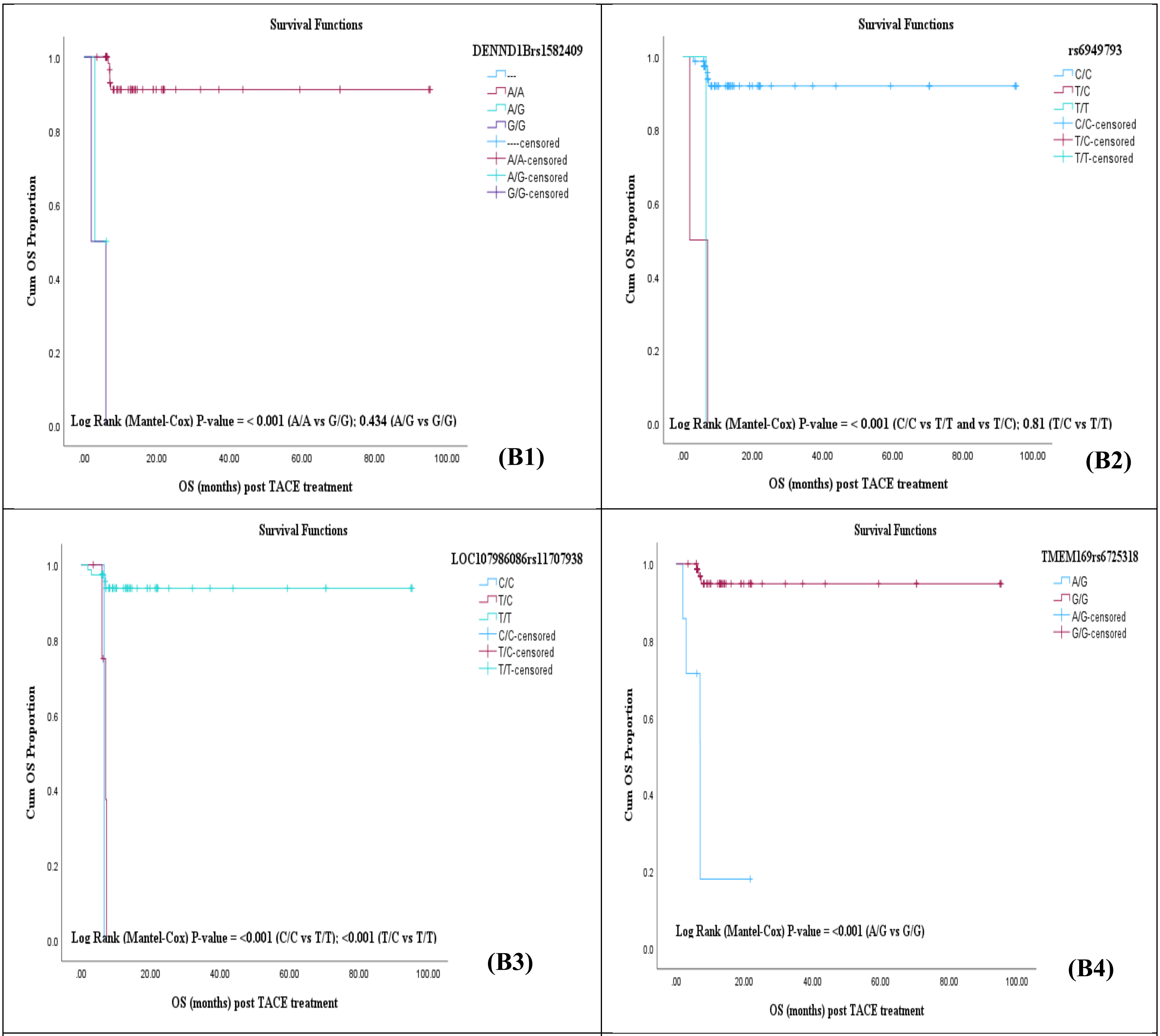

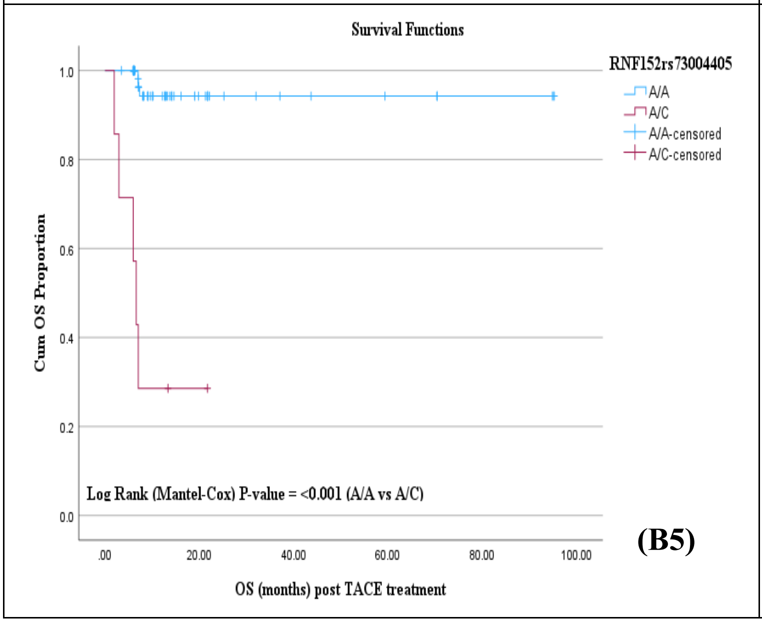

Supplementary Figure 3: Kaplan–Meier analysis of overall survival (OS) in HCC patients receiving doxorubicin chemotherapy mediated through TACE. (B1) OS in patients with different DENND1B rs1582409 genotypes. **(B2)** OS in patients with different rs6949793 genotypes. **(B3)** OS in patients with different LOC107986086 rs11707938genotypes. **(B4)** OS in TMEM169 rs6725318 genotypes. **(B5)** OS in patients with different RNF152 rs73004405 genotypes.
