# Supplementary figures and images for: Genetic polymorphisms as predictors of the response of hepatocellular carcinoma patients to doxorubicin chemotherapy: a genome-wide association study
Source: Front Pharmacol. 2025 Jun 4;16:1604473. doi: 10.3389/fphar.2025.1604473 (PMC12174396; doi:10.3389/fphar.2025.1604473)

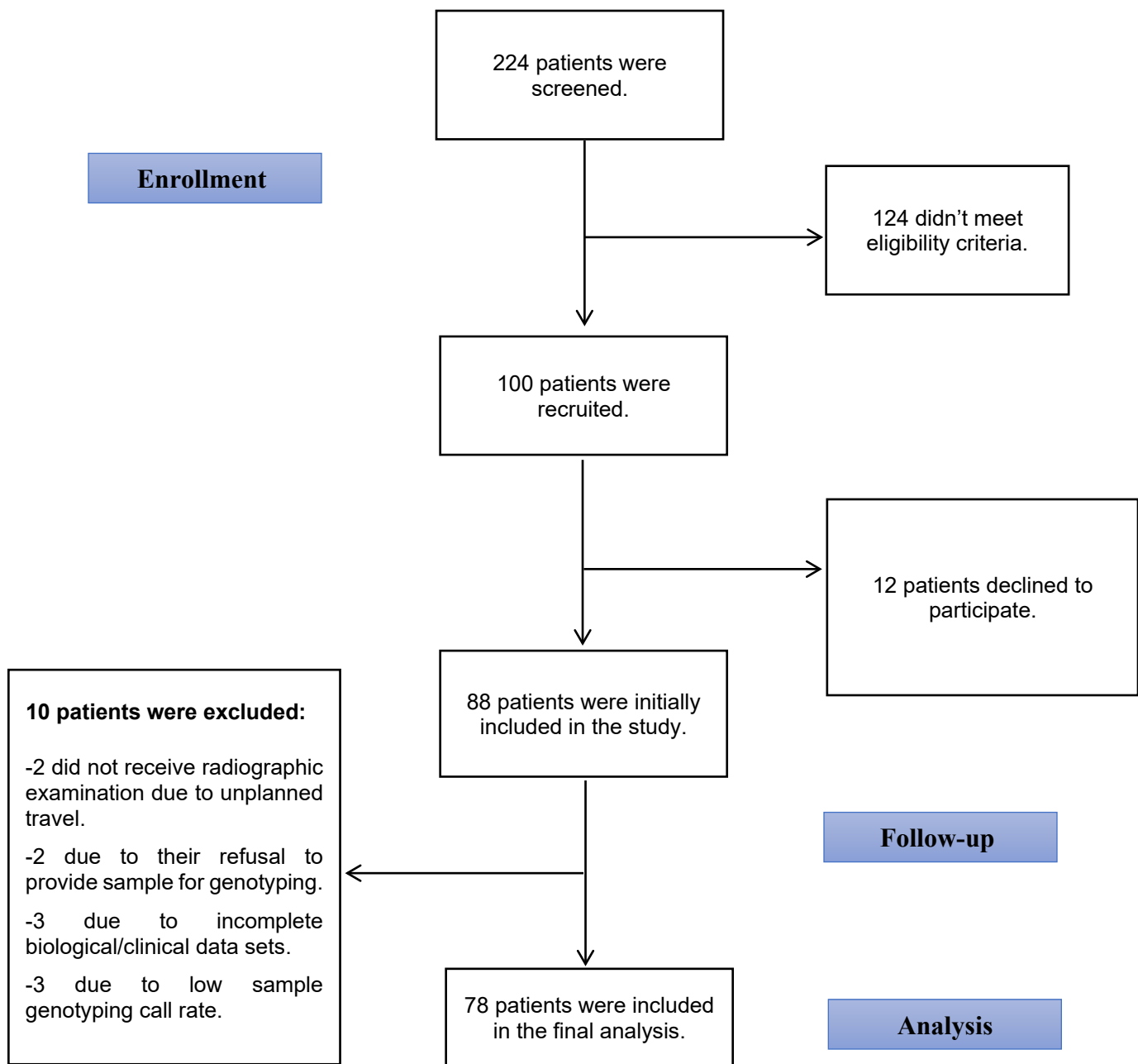

**Supplementary Figure 1: Consort Flow Diagram for patients' enrollment and follow-up.**

Supplement: Supplementary file 6 [file Image1.pdf]
